# Targeting Herpetic Keratitis by Gene Therapy

**DOI:** 10.1155/2012/594869

**Published:** 2012-12-26

**Authors:** Hossein Mostafa Elbadawy, Marine Gailledrat, Carole Desseaux, Diego Ponzin, Stefano Ferrari

**Affiliations:** ^1^The Veneto Eye Bank Foundation, Via Paccagnella 11, Padiglione Giovanni Rama, Zelarino, 30174 Venice, Italy; ^2^Cellectis Therapeutics SAS, 8 rue de la Croix Jarry, 75013 Paris, France

## Abstract

Ocular gene therapy is rapidly becoming a reality. By November 2012, approximately 28 clinical trials were approved to assess novel gene therapy agents. Viral infections such as herpetic keratitis caused by herpes simplex virus 1 (HSV-1) can cause serious complications that may lead to blindness. Recurrence of the disease is likely and cornea transplantation, therefore, might not be the ideal therapeutic solution. This paper will focus on the current situation of ocular gene therapy research against herpetic keratitis, including the use of viral and nonviral vectors, routes of delivery of therapeutic genes, new techniques, and key research strategies. Whereas the correction of inherited diseases was the initial goal of the field of gene therapy, here we discuss transgene expression, gene replacement, silencing, or clipping. Gene therapy of herpetic keratitis previously reported in the literature is screened emphasizing candidate gene therapy targets. Commonly adopted strategies are discussed to assess the relative advantages of the protective therapy using antiviral drugs and the common gene therapy against long-term HSV-1 ocular infections signs, inflammation and neovascularization. Successful gene therapy can provide innovative physiological and pharmaceutical solutions against herpetic keratitis.

## 1. Introduction

Gene therapy is the experimental use of genetic manipulation techniques to correct errors associated with genetic diseases or to modify undesirable Deoxyribonucleic acid (DNA) sequences. The ever extending list of genetic diseases opens the door wide to gene therapy as a new hope for targeting the aetiology rather than the symptoms of these diseases. Plenty of disciplines of gene therapy are currently discussed in the literature. However, there is a general agreement on few main issues to be thoroughly addressed before commencing a clinical trial for a novel gene therapy. Those include the precise diagnosis of the addressed genetic error, the relation between the causative gene defect and the resultant disease, the specific targeted tissue in the body, the dosage form design, and the choice of route of administration. Gene therapy approaches to corneal pathological disorders are being studied extensively to provide much needed progress against specific corneal malfunctions. Unlike protein based therapy, gene therapy has more research-attractive benefits being cheaper, better controlled, and more efficient in many occasions.

Herpes simplex virus type 1 (HSV-1) is a widespread human pathogen that causes life-long recurring disease. Two HSV serotypes exist, HSV-1 and HSV-2, with distinct tropism reported for each. The cold sore virus (HSV-1) is a leading cause of corneal blindness [1] and rejection of corneal grafts in the developed world [2]. The worldwide seroprevalence rates of HSV-1 ranges from 50 to 90% [3]. Most of the population acquires the infection during early age and adolescence. Both serotypes are reported to infect the cornea and the neural cells too. However, the vast majority of HSV infections in the eye are caused by HSV-1 serotype. Soon after the HSV-1 infection, the virus develops lifelong latency in the sensory ganglion of the trigeminal nerve (trigeminal ganglion) as confirmed in several reports [4–7]. When active, HSV-1 travels from the trigeminal ganglion to different destinations, including the cornea. However, there is a growing body of evidence suggesting that the virus may be able to remain in its quiescent stage within the stroma [8–10]. Acyclovir and corticosteroids are normally prescribed either alone or in combination for common HSV keratitis [11]. For example, prednisolone drops with prophylactic oral antiviral drug is common in stromal keratitis. The treatment of recurrent epithelial keratitis includes cycloplegia as well as antiviral eye drops such as trifluridine. 

As of January 2012, the online record gene therapy clinical trials worldwide provided by the Journal of Gene Medicine (http://www.abedia.com) shows 28 currently active clinical trials addressing different ocular diseases, such as age-related macular degeneration, choroideraemia, and glaucoma [12]. Interestingly, recent progress has been made targeting HSV-1 genome, as a new anti-viral class of medications [13, 14]. In this article, we review the current situation of ocular gene therapy against HSV-1 infections, including viral and non-viral vectors, routes of delivery of therapeutic genes and targets by screening and analyzing publications on ocular gene therapy from the published literature, and identify promising pathways, new techniques, and crucial research ideas. We also examine the advancements and prospects of ocular gene therapy, the progress and inadequacies, with potential solutions in this field of research. Targeting the genome of HSV-1 is a promising novel strategy in gene therapy field, and is the focus of this paper.

## 2. Background

### 2.1. Biology of HSV-1

**Figure 1:**
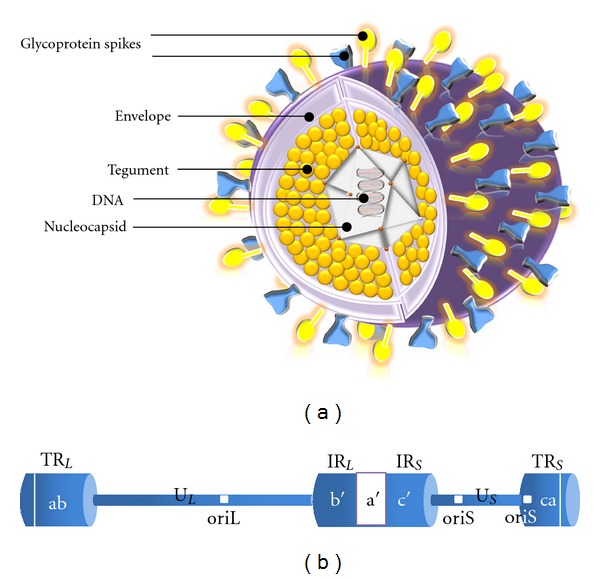
HSV-1 model structure and genome arrangement. (a) The icosahedral, DNA-containing capsid is asymmetrically located within the virion and surrounded by an amorphous protein layer called the tegument, and a membrane envelope heterogeneously studded with morphologically distinct spikes formed by 12 different glycoprotein species. (b) The HSV-1 genome arrangement showing repeats surrounding U_L_ designated ab and b′a′, and those surrounding U_S_ designated a′c′ and ca. There are two different origins of replication, oriL in the long segment and oriS in the short segment. Abbreviations: U_L_: long unique sequence; U_S_: short unique sequence; TR_L_: terminal repeats of long segment; TR_S_: terminal repeats of short segment; IR_L_: internal repeat of the long segment; IR_S_: internal repeat of short segment.

HSV-1 belongs to the human herpes virus (HHV) family. The HSV-1 virion is 120–300 nm in size. The genetic material of HSV-1 comprises 152,000 base pairs (encoding more than 80 genes) arranged as a double-stranded DNA, which circularizes upon infection [15]. The genetic material is surrounded by a capsid of a 100 nm diameter, a tegument and an envelope. The capsid of the HSV-1 virus is unique; the icosadeltahedral capsid is composed of 162 capsomers, surrounded by an amorphous structure containing various proteins and enzymes. The tegument surrounding the capsid contributes to the virulence of the virus. The virion is surrounded by a lipid bilayer envelope, acquired from the host, with 12 embedded glycoproteins. These glycoproteins function as attachment, fusion, structural and anti-immune proteins (Figure 1(a)). Five of these glycoproteins (gB, gC, gD, and the complex of gH and gL) mediate the entry of HSV-1 into host cells. Three dimensional structures revealed using cryoelectron tomography showed that the tegument was asymmetric where the capsid was more close to the envelope from one side. On the other side, they were distanced by *∼*35 nanometers of tegument [16]. The HSV-1 genome consists of a linear, double-stranded DNA molecules of 152 kb containing more than 80 genes and is composed of long (L) and short (S) unique (U) sequences regions (U_L_ and U_S_ resp.), which are flanked by regions of internal and terminal repeats. Those unique sequences are combined with specific origins of replication for each. Those repeats includes terminal repeat (TR) of long (TR_L_) and short segment (TR_S_), internal repeat (IR) of the long segment (IR_L_) and short segment (IR_S_). The sequences surrounding *U*_*L*_ region are labelled ab and b′a′ while those around the U_S_ region a′c and ca, see Figure 1(b). Around half of the genome sequences are conserved; however, mutations in other half of the genes have been reported not to affect the replication process. HSV-1 genome also contains a pac signalling sequence, essential for packing the DNA into the viral capsid [1]. 

### 2.2. Latency Mechanisms

During the latency stage of the HSV lifespan, the expression of latency-associated transcripts (LATs) is switched on, while general gene expression is restricted. Exit of the dormancy state can be triggered by decreased immunity due to infection, stress, ultra violet (UV) radiation or fever. The conjunctiva is believed to be the first affected organ by active HSV-1 infection [1]. Viral replication in the trigeminal ganglia continues until CD8+ (Cluster of differentiation 8) T-cell activity increases and the DNA mending mechanism cause the latency of the virus [17, 18].

### 2.3. Diagnosis of the Infection

Regarding the fact that asymptomatic HSV-1 infection is widespread, the diagnosis of HSV-1 through clinical symptoms is questionable. Laboratory methods performed by a specialized virologist is highly recommended [19]. The commonly used methods includes direct fluorescence antibody test, polymerase chain reaction (PCR) and cell culture using Vero cell line. However, direct fluorescence antibody test was reported to have superior sensitivity than PCR. Both methods had better sensitivity than the cell culture method, however, PCR showed higher degree of specificity [20]. Using antibodies against HSV-1 is a highly recommended method [21]. Recently, another group has shown that PCR and fluorescence antibody test have equal sensitivity: comparing specimens taken from cornea (by scraping) or from the patient's tears, results from PCR and fluorescence antibody test had similar sensitivity and negative predictive figures [22].

### 2.4. Clinical Management

Treatment of HSV-1 cases typically combines medications such as inflammation, immune and neovascularisation suppressing agents together with an anti-viral drug. Corticosteroids are used to improve clinical signs due its anti-inflammatory and antiangiogenesis effects. Several antiviral drugs have shown efficacy in the treatment of ocular HSV-1 keratitis, including acyclovir, valacyclovir, cidofovir, trifluorothymidine, and ganciclovir [23–25]. First line therapy for HSV-1 is acyclovir or valacyclovir [1, 26, 27]. Epithelial infection is usually treated with 3% acyclovir ointment for two weeks, while stromal necrotizing or non-necrotizing infections are treated with acyclovir in combination with a corticosteroid [28, 29]. Yet, resistance to acyclovir has recently been reported [30–32]. Therefore, novel antiviral drugs are highly demanded. Ganciclovir (as 0.15% gel) activity was established in the first case series describing effective treatment and prophylaxis of herpetic keratitis [33]. The commonly prescribed antiviral and corticosteroids drugs only alleviates the symptoms and shortens the infection period, but it does not prevent the reactivation of latent infections, consequently raises the demand for a new class of innovative powerful and safe genetic antiviral drugs. However, long-term administration of Acyclovir can help decrease the recurrence of the infection. Although few studies are discussing the possibility of producing a vaccine against both HSV serotypes [34, 35], the potential of treating ocular diseases using gene therapy is now more feasible. Gene therapy has the advantages of prolonged therapeutic effect, tissue-type specificity, transcriptional control via specific regulatory elements [36]. 

### 2.5. Immune Response to HSV-1 Infection

Understanding the mechanisms by which HSV-1 can survive or overcome the host immunity is a gateway for the development of novel antiviral therapies. Primarily, complex anti-viral defence mechanisms based on innate and adaptive immunity activates immune recruitment mechanisms. An immune cascade, orchestrated mainly by T-cells, is responsible for the pathogenesis of HSV-1. Other antiviral resistance molecules, grouped under “intrinsic antiviral immunity”, have recently emerged as a promising class in virus-battling research field [37]. Viral replication and assembly can be directly interrupted by preexisting agents in the host cell. Intrinsic factors, such as restriction factors, directly impede the normal viral lifespan [38].

## 3. Gene Transfer Vectors for Corneal Gene Therapy

Three approaches of gene transfer have been considered. The first one is systemic administration, even if the wide spread distribution in the host body is a major concern. Moreover, the gene delivery to the trigeminal ganglion where the HSV-1 latency requires high specificity and crossing the blood brain barrier to nerves. Second is the local application of naked therapeutic gene or a loaded vector. The third approach is to treat the cornea *ex vivo *where corneas can be treated prior to surgery. The main advantage here is safety and accessibility to the endothelium, even if the gene transfer can be performed only once.

The vectors must be chosen and modified to safely and efficiently escort DNA from outside the cell to the nucleus and to overcome several physical barriers that are obstacles to internalization, escape from endocytotic vesicles, movement through the cytoplasm, and transport into the nucleus. Vehicles for ocular gene therapy have been described in a broader prospect in number of reviews, therefore is not the focus of this paper. Nonetheless, here we attempt to fill the gaps, concentrate on the latest advances, and set up future directions for each vector type that can be used for corneal gene therapy of corneal herpetic keratitis. 

Most clinical trials for ocular gene therapy utilized viral vectors for gene delivery [12]. No record exists for clinical trials on herpetic keratitis gene therapy. Four classes of viruses are reportedly used in ocular gene therapy; those are adenovirus, adeno-associated virus, retrovirus, and lentivirus vectors. Nevertheless, the use of adenovirus and retrovirus was limited due to the relatively high inflammatory reactions of these viruses, plus their inability to transduce the all layers of the cornea, especially the endothelium [39].

**Adenovirus**

*Ex vivo* transduction of human corneas with adenoviral vectors containing lacZ reporter gene under either cytomegalovirus (CMV) or Rous sarcoma virus (RSV) transcriptional promoters provided high transduction efficiency especially in the endothelium [40]. Various approaches have been developed to modify adenovirus tropism. Changes have been introduced to adenovirus vectors to lower their immunogenicity. For example, the modification of the capsid protein polymers such as polyethylene glycol (PEG) extends circulation kinetics in murine models to allow neutralization of the adenovirus vectors by antibodies. The activated PEG reacts preferentially with the *ε*-amino termini of lysine residues on the capsid, specifically the hexon, fiber, and penton base proteins, resulting in improved specificity and lowered toxicity [41]. Moreover, the incorporation of a laminin derived peptide to a polymer modified adenovirus can improve its cell-type targeting specificity [42]. Several modifications can be introduced to adenovirus to improve its performance in gene therapy, approaches are discussed in a recent review [43]. Also adeno/HIV (human immunodeficiency virus) or adeno/AAV (adeno-associated virus) hybrids have been developed [44–46].

**Adeno-Associated Virus**

The current leading choice for corneal gene therapy is the adeno-associated virus (AAV). The expression pattern of this virus shows a delayed expression of the transgene; therefore, the therapeutic activity can be sustained for many years [47]. Recombinant AAV was successfully used to deliver therapeutic molecules to whole-thickness rabbit and human corneas *ex vivo* [48, 49]. Gene therapy using AAV vector to decrease corneal neovascularisation associated with corneal HSV-1 infections is reviewed elsewhere [39]. Recently, novel modifications to the viral genome and capsid were introduced to optimise the efficiency and tropism of AAV vectors. The genome of the new generation of self-complementary AAV vectors contains a covalently bound hairpin to form a duplex DNA molecule. The power point here is that self-complementary AAV vectors are no longer dependent on the host cell to convert the single-stranded AAV genome into transcriptionally active double-stranded forms. Self complementary AAV has shown a shorter lag time and believed to be a safer choice [50–53]. Additionally, the production of hybrid “pseudotyped” rAAV vectors, where rAAV genome is packaged in a capsid of another AAV serotype is verified in many reports and can improve tissue specificity and reduce toxicity and inflammatory reactions [54, 55]. The outcome of this mix and match process will not be reviewed here, as it is covered by others [54–56]. New comparative studies between different AAV serotypes for the transduction of human corneas (*ex vivo*) using intra-stromal injection was recently described [57]. Adeno/AAV hybrid vectors were shown to promote site-specific integration to avoid the activation of oncogenes after the integration of the transgene into human genome [44, 45].

**Lentivirus and Retrovirus**

Lentivirus and retrovirus belong to the group *Retroviridae*. Lentivirus based vectors is a popular choice for gene delivery to the endothelium, as it is known to proficiently transduce slow dividing cells. This class of viral vehicles has been shown to competently transduce primary human endothelial cells with 30% efficiency [58]. However, this property renders them unsuitable for stromal herpetic keratitis. On the contrary, successful lentivirus short hairpin RNA (ribonucleic acid) delivery to the cornea was used in a recent study, where a decrease in HSV-1 induced angiogenesis in stromal keratitis was observed [59]. Additionally, lentivirus was used for conveying therapeutic genes to improve corneal allograft endurance *ex vivo* [60].

The transduction efficiency of AAV can be improved by several methods, for example, creating a pocket with 110 *μ*m depth using femtosecond laser was shown to improve the efficiency of the lentivirus transduction of the stromal keratocytes, which is a promising method for antiangiogenesis agents that can improve the vision quality for HSV-1 after corneal transplantation [61].

Retrovirus (and lentivirus) genome is single stranded RNA, encapsulated in a lipid envelope. The name retro comes from its property of retro-transcription in linear double strand DNA to integrate into the host genome. Retroviral vectors recommended for transducing the cornea are based on different lentiviruses like HIV, equine infectious anaemia virus (EIAV) or feline immunodeficiency virus (FIV). The ability of retrovirus to transduce human corneas was confirmed in several report [62, 63]. Yet, they are not able to infect slow or non-dividing cells, hence cannot be used for endothelium transduction [64]. On the other hand, micro-RNA was successfully transduced in corneal epithelial cells as recently reported [65].

Retroviral vectors are well known to integrate their genome into the host to achieve stable transgene expression. This property is useful when treating chronic HSV-1 infection because of the long lasting gene expression benefit. Genes can be delivered to the endothelium by injection into the anterior chamber of the eye. Most retroviral vectors are genetically modified to isolate the *cis* sequences, required for the transfer of viral genome, from the *trans* sequences that encode viral proteins to produce replication incompetent vectors [66]. However, if recombination events generate a replicative virus, it is a main safety concern due to the pathogenic nature of HIV.

**Naked DNA**

Neovascularisation resulting from HSV-1 immuno-stimulation causes severe vision opacity. An early study showed that the transfer of naked complementary DNA (cDNA) encoding the vascular endothelial growth factor (VEGF) receptor antagonist to the eye was shown to block the formation of blood vessels [67]. DNA has been shown to access the epithelium and stroma to be transcribed into angiogenesis-controlling drug [68]. Topical application of a DNA plasmid encoding interferon alpha 1 (IFN-*α*1) was effective against ocular HSV-1 neovascularisation in a time and dose dependent manner, and able to antagonize HSV-1 reactivation [69].

**Microinjection**

Intraocular injection of DNA plasmid encoding Interleukin 18 (IL-18) reduced angiogenesis and immunoinflammatory lesions resulting from HSV-1 infection of mice [70]. Delivery to the cornea by microinjection into different corneal tissue was described in several research reports [71–73]. Polylactic coglycolic acid (PLGA) nanoparticles were shown to be efficient, nontoxic, and sustainable form for gene therapy, progressively reducing murine [74] and rat [75] corneal neovascularisation.

**Nanocarriers**

Nanoparticles are used in many drug delivery systems. Hyaluronic acid and chitosan nanoparticles can be loaded with genetic medicines, RNAi or DNA plasmids and used for corneal gene therapy. Albumin nanoparticles were able to reduce neovascularisation by 40% after 5 weeks, with no significant toxicity [71]. Controlled release silica nanoparticles impregnated with antiviral drug against HSV-1 (acyclovir) were shown to be a promising approach, however, it was only tested in biosynthetic corneal transplants [76], and it is suggested to design a similar dosage form for gene therapy delivery to the cornea. Copolymers of poly lactic and glycolic acids (PGLA) nanoparticles are proved to be a good vehicle for gene therapy [74], and low molecular weight chondroitin sulphate/hyaluronan nanoparticles were shown to competently deliver a Green fluorescent protein (GFP) encoding plasmid to rabbit cornea [77].

**Electroporation**

Electroporation method employs high field strength, square-wave electric pulses to allow the penetration of therapeutics molecules. Genetic material or therapeutic molecules are applied to the surface of the cornea, and then the electric pulse will assist the diffusion process. While this method does not involve any biochemical agents and is therefore considered a safer choice, electroporation can cause severe cell damage and induce immunogenic reactions [78, 79]. Alternatively, ultrasound can enhance gene transfer to mammalian corneal cells *in vitro* and *in vivo* without cell damage [80–82], and facilitates viral [83] and non-viral [84] gene and conventional drugs [81] delivery to the cornea. Electroporation was successfully used to deliver IL-10 gene to mice corneas [85]. *In vivo* gene transfer to the endothelium [86] and stromal keratocytes [87] has been reported in rats. Nevertheless high voltage (more than 500V) can be hazardous.

**Femtosecond Laser**

Applications of the femtosecond laser are becoming more accepted in corneal refractive surgery and transplantation, due to high precision and safety, compared to conventional laser. The femtosecond laser can be used to create a pocket where in the corneal surface to assist the delivery of therapeutic agents. It was used to deliver the vector to the stroma [61] therefore is suggested to be used in chronic stromal herpetic keratitis conditions, if the latency of HSV-1 is in the stroma, as recently argued [9]. The femtosecond infrared titanium sapphire laser beam was shown to enhance *in vivo* gene delivery [88]. This method has been shown to effectively assist the closure of corneal neovascularisation in rabbits [89].

**Synthetic Peptide Vector Systems**

Antibody targeting can be a useful addition to the liposome gene transfer techniques. The use of antibodies with certain cell-specificities can be useful to target a specific corneal layer. Liposomes containing plasmid DNA and coated with antibodies will form an immuno-specific vector to target specific receptors of a given cell type, for example, targeting the endothelium to improve allograft survival [90, 91]. Liposomes and transferrin (targeted to endocytic transferrin receptor) have been used successfully to modulate murine corneal allograft rejection with a therapeutic transgene. A vector system based on synthetic peptides of polylysine and molossin has been shown to deliver DNA plasmid to all rabbit cornea endothelial cells after direct application *ex vivo* [92].

**Gene Gun**

Gene gun method is also known as particle bombardment, microprojectile gene transferor gene gun. Ballistic transfer with the gene gun can be used to transfer cDNA coated gold particles to the epithelium [93]; however, the use of gene gun to transduce the endothelium was shown to severely damage [94].

**Dendrimer and Liposome-Based Delivery**

Dendrimers are macromolecules characterised by extensively branched three dimensional structure that can accommodate a genetic material, hence used for drug delivery. Activated polyamidoamine dendrimers carrying a DNA plasmid transduced up to 10% of corneal endothelium after direct application to the cornea [95]. Liposome gene transfer to the endothelium was reported [96]. The relative inefficiency of these vectors has limited their use in corneal gene therapy. However, polyethylene glycol-modified liposomes or the aid of ultrasound can improve the efficiency for drug delivery.

## 4. Gene Therapy Strategies

A body of work in gene therapy for corneal disorders has established the potential of finding new anti-HSV-1 gene therapy. Drug delivery to the cornea by gene therapy is preferably applied locally to avoid systemic complications, or performed *ex vivo* in an eye bank to improve the graft survival by prior to transplantation in a patient with herpetic keratitis history. Topical application may avoid modulation of the systemic immune response to HSV-1, and it is not likely to induce untoward corneal inflammation or systemic side effects. Several strategies have been used to target certain viral envelope component, replication process, protein, or the resultant inflammation. Most of the gene therapy work on herpetic keratitis was targeting the chronic inflammation process; however, less significant amount of data exists on targeting the virus genome.

### 4.1. Gene Therapy Targeting Inflammatory Mediators or Neovascularisation

Most of the gene therapy approaches to combat HSV-1 were directed to the inflammation process. In 20% of herpetic keratitis infections, when the latent virus is reactivated it results in chronic ocular immune response where specific lymphocytes are produced, mainly CD4^+^ T producing T-helper 1 (Th1) cytokines [97]. Herpetic stromal keratitis chronic inflammation causes edema, neovascularisation and scarring. Moreover, 5–10% of cornea transplantation operations is performed to replace HSV-1 damaged corneas [98]. Immunogenicity from herpetic keratitis infection can be reduced; however, reducing host sensitization is more preferable. A reasonable number of studies have been exploring the ability of gene therapy to deliver specific anti-inflammatory mediators, as briefed in Table 1, where successful attempts to target ocular HSV-1 by gene therapy are arranged in chronologic order to highlight the progress achieved. Reducing the inflammation and/or the neovascularisation will result in the desired regression of the herpetic keratitis signs. Additionally, survival rates of corneal allograft can also be improved. Nevertheless, it must be considered that the disease was masked and not actually cured. Therefore, the interest in the recent years was directed to the viral genome itself, as detailed in Section 4.2.

### 4.2. Gene Therapy Targeting the HSV-1 Genome

DNA damage response is a mechanism by which cells can correct damage or eliminate severely damaged cells by activating programmed cell death mechanisms. DNA damage mechanisms are involved in processes such as excision of the damaged area, cell cycle arrest to prevent the pass on of mutated sequences or the transcriptional level control. However, severe injuries can cause the cell to undergo apoptosis. The DNA repair mechanisms include direct repair, base excision repair, nucleotide excision repair, double-strand break repair, and cross-link repair. Detailed description of these mechanisms is discussed elsewhere in more detail [125]. Understanding these mechanisms allows for the discovery of new genetic agents as possible antiviral candidates.

**Figure 2:**
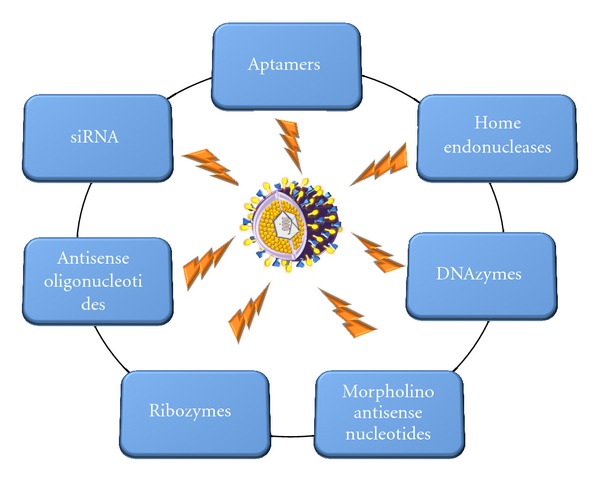
Successful targeting of HSV-1 genome by gene therapy to date. Abbreviations: DNAzymes: deoxyribozymes; siRNA: small interfering RNA; HSV-1: herpes simplex virus serotype 1.

Targeting the HSV-1 genome for degradation, using several techniques have been reported, including the use of ribozymes [126], antisense oligodeoxy nucleotides [127], morpholino antisense nucleotides [128], small interfering RNA (siRNA) [117], aptamers [129], and homing endonucleases [130] (Figure 2).

**Ribozymes**

Ribozymes are RNA molecules with intrinsic enzymatic activity to promote a variety of reactions without the aid of protein cofactor, usually involving cleaving or splicing of RNA molecules, therefore, can be used for gene therapy [131]. They have been shown to be active against HIV-1 [132] and HSV-1 [126, 133], where it improved the survival rate in mice. Ribozymes are less potent than siRNA, however, the evolution of new ribozymes and the relatively better specific binding are two advantages.

**Antisense Oligodeoxynucleotides**

Antisense oligodeoxynucleotides are short synthetic DNA consisting of 15 to 20 nucleotides. They inhibit protein biosynthesis by specifically targeting the complementary stretches of RNA. In principle, they are able to interfere with each step of nucleic acid metabolism, preferentially to block translation [134]. Antisense oligonucleotides were used against HSV-1 [127, 135], moreover, topical treatment was found to reduce TNF-*α* in cultured lymphocytes and *in vivo* in mice, but systemic administration was not successful [114]. However, the topical TNF-*α* blockade may interfere with the antiviral response, ultimately leading to recurrent herpetic keratitis.

**Morpholino Antisense Oligonucleotides**

As antisense oligonucleotides, morpholinos function by translational arrest. Phosphorodiamidate morpholino oligomers (PMOs) are a subclass of antisense oligonucleotides modified to include a phosphorodiamidate linkage and morpholine ring to demonstrate limited off-target effects, favourable base stacking, high duplex stability, high solubility, cell permeability, and no hybridization complexities [136]. In a recent promising study, targeting the translation sites ICP0 in HSV-1 genome in mice with peptide conjugated PMO showed better inhibition than acyclovir [128].

**Small Interfering RNA (siRNA)**

The ability of transfected synthetic siRNAs to suppress the expression of specific transcripts is a useful technique to probe gene function in mammalian cells. Plasmids encoding small hairpin RNAs are extensively used in gene therapy. Consequently, siRNA was used to target viruses in several studies [137–139]. Targeting neovascularisation resulting from herpetic keratitis, Kim et al., [117] have shown that siRNAs against VEGFA, VEGFR1 and/or VEGFR2 reduces lesions and angiogenesis. Additionally, siRNA targeting glycoprotein E expression, which mediates cell-to-cell spread and immune evasion, was shown to suppress HSV-1 active infection *in vitro* [140]. Knock-down of glycoprotein D was also achieved using siRNA on HSV-1 infected human conjunctival epithelium *in vitro* [141]. Using *in vitro* plaque test, HSV-1 replication was also inhibited by siRNAs against the UL39 gene encoding the large subunit of ribonucleotide reductase, ICP6 [142]. Although siRNA based approaches are highly specific, they are yet still expensive, and not really reproducible due to variability in efficiency and transient expression nature of the technology.

**Aptamers**

Oligonucleotide sequences with the capacity to recognize specific target molecules with high affinity and specificity, referred to as “aptamers”, are beginning to emerge as a class of molecules that compete with antibodies in both therapeutic and diagnostic applications. Recent studies have shown *in vivo* inhibition of rat corneal angiogenesis by targeting angiopoietin-1 with nuclease-resistant RNA aptamers [129].

**Homing Endonucleases**

Targeting endonucleases to HSV-1 genome for excising is an emerging new concept for antiviral gene therapy. Homing endonucleases are a group of restriction enzymes encoded by introns and inteins. Their recognition sites are rare, however, custom made endonucleases, namely meganucleases, can be made targeting specific viral sequences for gene therapy [13, 14, 130, 143, 144]. Anti-HSV custom-made meganucleases were recently shown to prevent the infection of cultured cells by wild-type HSV-1 [130].

### 4.3. Gene Therapy for New Targets

Several viral components have been proposed as promising targets for antiviral drug discovery by targeting them in a highly selective and effective manner [145]. The delivery of genes encoding agents that are capable of disrupting numerous biological processes is a key concept. Targeting the reverse transcriptase can block the transcription process [146]. Viral surface receptors also represents attractive targets [147], for example, C-C chemokine receptor type 5 (CCR5) which is a major coreceptor important for efficient viral entry into cells. Additionally, a number of replication proteins serves as interesting targets for gene therapy, such as thymidine kinase UL23, ribonucleotide reductase UL39, 40, deoxyuridine diphosphatase UL50, uracil-DNA glycosylase (UL2), and alkaline nuclease (UL12) [148]. Other strategies include the inhibition of DNA polymerase by specific peptides or small molecules [149, 150], nuclear localisation signalling molecules [151], and the inhibition of protein-protein interactions [145], where a single substitutions in one subunit of a protein-protein interface can completely disrupt subunit interactions. For example, interruption of a few hydrogen bonds between subunits UL30 (Pol) and UL42 may represent an interesting target for new anti-HSV-1 drugs [149]. 

## 5. Future Direction

The potential in the treatment of many genetic diseases and viral infections is progressively elevating the attention and awareness towards this new era of highly specialized treatment. The fact that gene therapy is now being clinically evaluated, promotes the massive research that is conducted currently in animal models. It is well established that therapeutic genes or molecules can be transferred to the cornea by direct application of naked DNA, electric pulse, ballistic transfer with a gene gun, viral and non-viral vectors, or several creative combinations of these approaches. The choice of the appropriate vector for targeting HSV-1 is essential for successful gene therapy. The expression profile, bioavailability, biodegradability, and specificity of the vector are the main variables currently evaluated by several research groups to achieve the ideal combination. Successful gene therapy requires the choice of the right vector for each particular disease. Choices are made based on the ability of the targeted cells to uptake the vector, expression of the transgene in a cell-specific manner, and maintenance of expression for the period of time needed to tackle the disease. For chronic cases not requiring a surgery, local application to the cornea is usually preferred. However, pretreatment of corneas *ex vivo* in eye banks with specialised areas and trained staff is a new practical approach to corneal HSV-1 gene therapy. This can help decrease transplants rejection due to chronic herpetic keratitis and minimise the risk of *in vivo* gene therapy.

## Figures and Tables

**Table 1:** 

Chronologic order	Vector	Gene	Host	Rout of administration	Results and reference
1990	Vaccina virus	gD	Rabbits	Intradermal injection	No effect on herpes simplex keratitis during 16 days postinfection [99]

1997	Plasmid DNA	IL-10, IL-2, GM-CSF	Mice	Topical or intramuscular	Topical: IL-10, only, reduced lesion severity. Intramuscular: no effect [100]

1998	Plasmid DNA	IL-2, IL-4, IL-10, IFN-*γ*	Mice	Topical	IL-4 or IL-10: reduced lesion severity 25 days post-infection; IL-2 or IFN-*γ*: no effect [101]
	Plasmid DNA	IL-2, IL-4, IL-10	Mice	Topical, nasal, or intramuscular	Topical administration of IL-4 or IL-10 lowered lesion severity 21 days after infection [102]

1999	Plasmid DNA	IFN-*α*1	Mice	Topical	Increased survival if treated 1 day pre-infection [69]

2000	Plasmid DNA	gB1	Rabbits	Subconjunctival or intramuscular injection	Complete survival and reduction of lesions following intramuscular administration only [103]
	Plasmid DNA	gD, gD-IL-2	Mice	Subconjunctival injection	Good survival for grafts and prevention of stromal, but not epithelial, keratitis [104]
	Plasmid DNA	IFN-*α*1	Mice	Topical, nasal, or vaginal administration	Topical application improved the survival when treated 1 day before infection [105]
	Plasmid DNA	IFN-*β*	Mice	Topical	Increased survival only when applied 1 day before infection [106]
	Plasmid DNA	IFN-*α*1	Mice	Topical	Increased survival only when applied 12 hours before infection [107]

2001	Plasmid DNA	IFN-*α*1	Mice	Topical	Increased survival [108]
	Plasmid DNA	gB	Mice	Mucosal	Increased INF-*γ* [109]

2002	Plasmid DNA	gD-IL-2	Mice	Subconjunctival injection	Complete prevention of stromal, but not epithelial, keratitis 10 days after infection [110]
	Plasmid DNA	gD-IL-2	Mice	Topical conjunctival	Complete prevention of stromal, but not epithelial, keratitis [111, 112]
	Plasmid DNA	IL-12, IP-10	Mice	Topical	Suppression of lesions using both transgenes. IP-10, but not IL-12, suppressed lesions [113]

2003	Antisense oligonucleotides	TNF-*α*	Mice	Subepithelial injection	Reduction of herpes simplex keratitis signs 14 days after infection [114]
	HSV-1	IL-2, IL-4, IFN-*γ*	Mice	Intraperitoneal injection	All transgenes: complete survival and prevention of corneal scarring 28 days after infection [115]

2004	Plasmid DNA	gB, gC, gD, gE and gI	Mice	Intramuscular injection	Complete survival and prevention of corneal scarring 28 days after infection [116]
	siRNA duplexes with or without TargeTran	VEGF	Mice	Subconjunctival or intravenous injection	Subconjunctival or systemic administrations suppressed lesions 10 days after infection [117]

2005	HSV-1	IL-12p35, IL-12p40	Mice	Intraperitoneal injection	Both transgenes: complete survival and prevention of corneal scarring 28 days after infection [118]
	Plasmid DNA	IL-18	Mice	Topical	Suppression of lesions 12–21 days post-infection [70]
	Plasmid DNA encoding shRNA	Matrix metalloproteinase-9	Mice	Intrastromal injection	Suppression of lesions during 21 days after infection [119]

2008	siRNA	TNF-*α*	Mice	Intraperitoneally	Inhibition of TNF-*α* [120]
	Plasmid DNA	IL-10	Mice	Topical	Modulate the inflammation severity [121]

2009	Plasmid DNA	VEGFR2 VEGFR3	Mice	Subconjunctival injection	Control vascularisation after HSV-1 infection [72]

2010	Nanoparticles	IL-21 and gD vaccine	Mice	Ocular mucosal administration	Inhibits ocular HSV-1 [122]
	DNA plasmid	B and T lymphocyte attenuator	Mice	Intraperitoneal injection	Decreased CD4^+^ T mediated cell response [123]

Abbreviations—gD: HSV-1 glycoprotein D; GM-CSF: granulocyte-macrophage colony-stimulating factor; INF: interferon; IL: interleukin; IP-10: IFN-inducible protein 10; shRNA: short hairpin RNA; siRNA: small interfering RNA; TNF: tumour necrosis factor; VEGF: vascular endothelial growth factor. Table updated from [124].
